# Endophytic bacterial communities in peels and pulp of five root vegetables

**DOI:** 10.1371/journal.pone.0210542

**Published:** 2019-01-11

**Authors:** Viia Kõiv, Krõõt Arbo, Ülo Maiväli, Veljo Kisand, Märt Roosaare, Maido Remm, Tanel Tenson

**Affiliations:** 1 Institute of Technology, University of Tartu, Tartu, Estonia; 2 Institute of Molecular and Cell Biology, University of Tartu, Tartu, Estonia; Nicolaus Copernicus University, POLAND

## Abstract

Plants contain endophytic bacteria, whose communities both influence plant growth and can be an important source of probiotics. Here we used deep sequencing of a 16S rRNA gene fragment and bacterial cultivation to independently characterize the microbiomes of five plant species from divergent taxonomic orders—potato (*Solanum tuberosum*), carrot (*Daucus sativus*), beet (*Beta vulgaris*), neep (*Brassica napus spp*. *napobrassica*), and topinambur (*Helianthus tuberosus*). We found that both species richness and diversity tend to be higher in the peel, where *Alphaproteobacteria* and *Actinobacteria* dominate, while *Gammaproteobacteria* and *Firmicutes* dominate in the pulp. A statistical analysis revealed that the main characteristic features of the microbiomes of plant species originate from the peel microbiomes. Topinambur pulp displayed an interesting characteristic feature: it contained up to 10^8^ CFUs of lactic acid bacteria, suggesting its use as a source of probiotic bacteria. We also detected *Listeria sp*., in topinambur pulps, however, the 16S rRNA gene fragment is unable to distinguish between pathogenic versus non-pathogenic species, so the evaluation of this potential health risk is left to a future study.

## Introduction

During the past decade our knowledge about the microbes that inhabit higher organisms has increased considerably. In general, higher organisms contain a species specific core microbiome and a more random set shaped by the environment [1–4].

Plants acquire endophytes mainly from their roots and the surrounding soil biome, thereby making the plant microbiome highly dependent on the environment [5–9]. Recently it has been shown, that the mammalian diet has strong influence on their gut microbial communities [10–12]. While it is well documented how the chemical composition of food—fibers, sugars, and fat—influences bacterial richness in human gut by suppressing or enhancing the growth of specific microbe groups [13–15], the contribution of the live bacterial part of our diet has been less intensively studied. Still, it is obvious that, by consuming food we introduce microbes into our digestive tract, the majority of which are eliminated by low pH in the stomach and are unable to compete with the resident microbiome in the digestive tract. Most of our knowledge about the transmission mechanisms of bacteria into our intestine are derived from the study of pathogen transmission [16], however, recent studies have extensively studied the ingested temporal colonization of lactic acid bacteria in the gastrointestinal tract [17–20].

To our knowledge, only one study has been published about the general microbial content in foods [21] and a few studies have focused on the contribution of microbes within vegetables on our food [22,23]. Research on the health effects of vegetable microbiomes has also largely focused on lactic acid bacteria on the beneficial side and on *Salmonella*, *Listeria*, and *Escherichia coli* on the pathogenic side, largely neglecting other microbial groups. There are many other studies on plant microbiomes; however, these do not specifically approach the consumption of the plants as food. The microbiomes of raw edible plants have mainly been characterized in tissues that live above ground such as lettuce [24], tomato [25], and grapevine [26,27]. For root vegetables, the microbiome has only been described in potato [28,29], carrot [30], and young radish [31]. Previously, we have observed rich and dynamic endophytic microbial communities in potato tubers during storage [32]. Taking into account WHO recommendations to increase the fraction of fruits and vegetables in our diet [33], there is good cause to further study the impact of consuming live vegetable microbiomes.

Plant microbiota is gradually enriched by the microbial community within soil [34]. The highest number and diversity of plant-associated bacteria has been observed in the rhizosphere [2,3]. From there, a number of researchers have observed a gradient in the microbiome from the surface to the inner parts of the root [2,3,35,36]. Thus, we expect that peeling root vegetables will have an influence on the microbial composition of the food. In this context, it should be emphasized that soil microbial communities have very strong potential for colonizing mammalian gut [37], which suggests that soil-like communities in the peel might have strong colonization potential in the gut.

Here, we analyze the microbiomes of five common root vegetables—potato (*Solanum tuberosum*), carrot (*Daucus sativus*), beet (*Beta vulgaris*), neep (rutabaga) (*Brassica napus spp*. *napobrassica*), and topinambur (Jerusalem artichoke) (*Helianthus tuberosus*)—which have been selected to represent five different orders of plants. In order to assess the effect of peeling on the microbiome composition, the microbiomes of peels and pulps were studied separately.

## Materials and methods

### Sampling

Root vegetables carrot (*Daucus sativus*) cultivar “Natalia”, beet (*Beta vulgaris*) cultivar “Modena”, neep (rutabaga) (*Brassica napus spp*. *napobrassica*) cultivar “Globus”, and potato (*Solanum tuberosum*) cultivar “Laura” were grown by organic farming in Võnnu, Tartumaa county, Estonia (58.17179, 27.04064), soil type leptosol (LP). For fertilization, we used NPK 11-9-20, Cropcare 11-11-21, and ALLGROW bioplasma. Jerusalem artichoke (topinambur) (*Helianthus tuberosus*), cultivar unknown, was grown in virgin soil in Vetiku village, Lääne-Virumaa county, Estonia (59.192367, 26.263193), soil type silt (M”‘). No specific permissions were required for these locations/activities, because the vegetables originated from routine production and no field studies were performed. The research did not involve endangered or protected species.

Carrots, beets, neeps, and potatoes were planted in May and harvesting in October 2014, kept until January 2015 in a common cellar at +5°C and subsequently refrigerated for two weeks at +4°C. Topinamburs were constantly grown in same place for 5 years, were harvested in January 2015 and kept at +4°C in a refrigerator for two weeks. Of each species, five vegetables of similar size and shape were chosen. Before peeling, the vegetables were carefully washed and brushed with dish washing liquid “Fairy” (Procter & Gamble), rinsed with tap water and thereafter kept in sterile distilled water for 10 minutes. For peel extraction, all vegetables were grated with a sterile grater, after which 7 grams of the peel were mixed with 7 ml of sterile 10 mM MgSO_4_ in 50 ml Falcon tubes. The inner tissues, i.e. the pulp of the grated vegetables, were obtained by making several cuts with a sterile scalpel to obtain an intact sample of the inner tissues. Seven grams of the pulp of these vegetables were cut into pieces, homogenized with a sterile garlic smasher, and mixed with 7 ml of sterile 10 mM MgSO_4_ in 50 ml Falcon Centrifuge Tubes. The tubes were alternately vortexed and kept on ice for 10 minutes, and then 1 ml of this suspension was transferred into 1.5 ml Eppendorf tubes. The remainder of homogenized vegetables was stored in the same Falcon tubes at -20°C. The suspension inside the Eppendorf tubes was centrifuged at 13 000 x g for 5 minutes (Heraeus Biofuge Pico, Germany), the supernatant was discarded and the pellet stored at -20°C until extraction of the DNA. For the second DNA extraction replicate, the frozen vegetables kept at -20°C were melted and the same procedure as described above was followed again.

### DNA extraction, PCR and sequencing

Our DNA extraction strategy involves two sequential extraction steps of increasing stringency to improve the coverage of the microbiome. In the first extraction, DNA was extracted using a RTPBacteria DNA Mini Kit (Stratec Biomedical Systems, Germany) according to the manufacturers protocol, with one additional step: the cells were lysed by bead beating with Zirconia/Silica beads (Biospec Products, USA): 0.1 mm– 0.5 g with FastPrep-24 (MP Biomedicals, USA) at 4 m/s for 3 x 60 seconds. The V4 region of the 16S rRNA gene was amplified using modified versions of the primers designed by Sakai et al. (2004). The PCR amplification was performed with primers F534ad (3’- GTGYCAGCMGCCGCGGTAAT -5’), R783ad (3’-ACVMGGGTWTCTAAKCCKG-5’) using Phusion High-Fidelity DNA Polymerase (ThermoFisher Scientific, USA) and approximately 20 ng of DNA in a 25 μl reaction mixture. The PCR reaction was carried out at 98°C for 2 min followed by 25 cycles each of 98°C for 20 s, 50°C for 30 s, and 72°C for 15 s, followed by 10 min at 72°C. PCR amplifications were performed in triplicate and then pooled. The pooled PCR products were cleaned using an UltraClean PCR Clean-Up Kit (MoBio, USA), and both the quantity and quality of DNA was determined spectrophotometrically (NanoDrop 2000c). Even though the obtained DNA had high yields and favorable quality measures according to NanoDrop measurements, PCR reactions were inhibited in several samples, especially those of turnip, carrot, and beet.

DNA sequencing was carried out using the Illumina MiSeq platform (San Diego, USA). A total of 578 498 high-quality reads spanning the V4 region of the 16S rRNA gene were obtained with a median read count per sample of 11 833 (range: 1 251–27 048). About 78% of the reads were classified as chloroplasts or mitochondria. The low number of bacterial reads prompted us to re-extract DNA from what was left of our original grated samples (frozen at -20°C).

In the second extraction, one additional step was added: before the purification step with the columns provided by RTPBacteria DNA Mini Kit (Stratec Biomedical Systems, Germany), the lysis mixtures were extracted once with chloroform. The chloroform extraction step was introduced to remove some plant compounds that inhibit the DNA polymerase reaction and considerably improved the PCR reaction. Another difference was that unlike in the first experiment, we used the original primer set by Sakai et al (2004) for the PCR amplification in the second experiment. Primers F534adnov (3’- CCAGCAGCCGCGGTAAT -5’) and R783adnov (3’-ACCMGGGTATCTAATCCKG-5’), Maxima Hot Start Taq DNA Polymerase (ThermoFisher Scientific, USA) and 30–40 ng of DNA in a 25 μl reaction mixture were used. The PCR reaction was carried out at 95°C for 5 min followed by 7 cycles each of 95°C for 45 s, 65°C for 60 s, and 72°C for 90 s, followed by 20 cycles of 95°C for 45 s, 50°C for 30 s, 72°C for 90 s and final extension 10 min at 72°C.

DNA sequencing was carried out using the Illumina MiSeq platform. This time a total of 7 249 842 high-quality reads were obtained with a median read count per sample of 147 738 (range: 7 996–230 517). Only about 3.3% of the reads were classified as chloroplasts or mitochondria.

All sequencing files are available from the European Nucleotide Archive (https://www.ebi.ac.uk/ena) database (accession number PRJEB30251).

### Sequence processing and clustering of 16S rRNA reads into OTUs

Firstly, raw paired-end reads of the resultant sequences from the two extractions were quality-filtered using Trimmomatic (parameters *LEADING*:*3 TRAILING*:*3 SLIDINGWINDOW*:*6*:*25 MINLEN*:*100*) [38]. Surviving pairs of two extractions were pooled. For read clustering and analysis, we used CD-HIT-OTU workflow for Illumina data (39). It includes the steps of paired-end reads merging and detecting chimeric sequences. The combined dataset was clustered into OTUs using >97% sequence identity. Two OTU abundance tables from the different extractions were created and chimeric sequences were removed using CD-HIT-OTU [39] (http://weizhongli-lab.org/lab-wiki/doku.php?id=cd-hit-otu-user-guide). Representative OTU sequences were identified using MegaBLAST (parameters *-p 90 -D 3 -v 5 -b 5 -W 44*) [40] and SILVA database (release SILVA 123; SSU Ref Nr 99) [41], taking the hit with highest bit-score as the representative organism for this OTU.

In order to exclude sequences observed at very low frequencies, OTUs representing less than 0.001% of the total number of sequences from each library were removed. OTUs classified as mitochondria or chloroplasts were discarded from the OTU table.

### Cultivation

Dilutions of the melted samples for CFU counting were plated on R2A agar (LabM) and FAA (LabM), the plates were incubated for one week at room temperature. At least three replicate cultivations were made from all frozen samples. The dominant bacteria were identified using Matrix Assisted Laser Desorption/Ionization Time of Flight instrument (MALDI-TOF; Bruker Daltonik GmbH, Germany; Instrument ID: 269944.00686). The Biotyper software was then used to identify bacterial species. The Real Time identification score criteria used were those recommended by the manufacturer: score ≥ 2.000 indicates species-level identification, score ≥1.700 and <2.000 indicates genus-level identification and a score <1.700 indicates no reliable identification. Run Identifier 161208-1412-1019.

### Statistical analyses

Statistical analyses were performed in R 3.3.2 [42]. OTU counts derived from sequencings of the two DNA extractions were summed in order to make a joined OTU matrix. We discarded the OTUs whose read counts in all samples were less than ten from further analyses. For indicator species analysis and detailed analyses of most abundant phyla / classes of vegetable microbiomes (S1 Fig), we included only OTUs which read counts in all samples were more than 50. For the alpha-diversity measurement, Shannon’s Diversity and Chao1 richness were calculated with the vegan::diversity function [43]. Significant differences were determined via Tukey’s post hoc test in R [44]. For beta-diversity analyses (i.e., PERMANOVA, CAP), normalization was carried out by summing the proportion of OTU counts derived from the two extractions. Constrained multidimensional scaling using the Constrained Analysis of Principal Coordinates (CAP) was performed using the vegan::capscale function [43]. For permutational multivariate analyses of variance (PERMANOVA), we used the vegan::adonis function [43]. The core microbiome was identified using the compute_core_microbiome.py script from the QIIME package [45]. The indicator species were studied using the R function indicspecies::multipatt [46]. Relative abundances of OTUs specific to only a single vegetable (P < 0.001) were clustered using Bray-Curtis dissimilarity [47] and depicted on a heatmap using the gplots::heatmap.2 function [48].

## Results

To characterize the microbial communities of root vegetables, we used potato, neep, beet, and carrot harvested in 2014 from organic agriculture fields around Võnnu, Estonia, and topinambur from an organic agriculture field around Vetiku, Estonia. The vegetables were stored until January 2015. In total, we obtained five samples per species. The endophytic bacteria of vegetables were isolated separately from peels and pulp. The vegetable material was homogenized and DNA was extracted. As opposed to extraction of total DNA of the unfractionated sample, this approach allows us to reduce the fraction of plant chloroplast and mitochondrial sequences. Two complementary DNA extractions were used and data were combined, allowing for more complete characterization of the microbiome.

### Diversity of root-associated microbiomes

**Fig 1:**
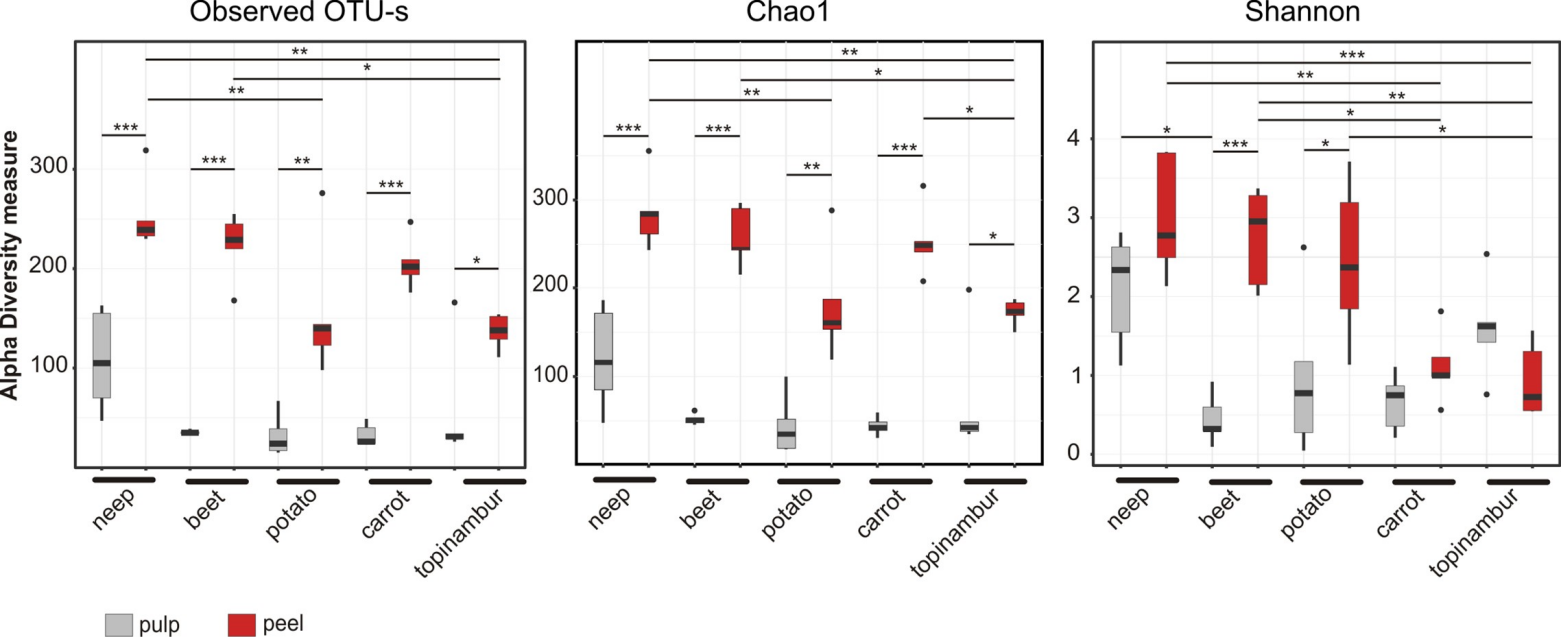
Estimates of the alpha diversity of the bacterial communities in root vegetables. Richness is based either on the number of observed OTUs (Observed OTU-s) or the estimated Chao1 index (Chao1) and the diversity is based on the Shannon index (Shannon). Significant differences between peels and pulps (following the Tukey post hoc test) are indicated at the top of each box: *** P < 0.001; ** P < 0.01; * P < 0.05.

To gain insight into the microbiota richness and α-diversity of the vegetables, we used 16S rDNA sequencing and the reads were clustered using >97% sequence identity into 816 microbial OTUs. In all vegetables, the peel had a significantly higher number of OTUs than the pulp (Tukey post hoc test P < 0.05). The number of OTUs inside the neep (median 105) was higher than in other vegetables (all median values below 40) and comparable with the OTU count in the peel of potato (median 140) and topinambur (median 138). The median numbers of OTUs in the peel of beet, carrot and neep were around 220. The OTU counts are in accordance with the estimated richness (Chao1), which reflects the theoretical richness of OTUs (Fig 1).

According to the Shannon index, which accounts for both microbial richness and abundance, microbiota of neep, beet and potato peels tend to exhibit higher diversity than their pulp (Fig 1). Interestingly, the Shannon index indicates substantially lower microbial diversity within the peels of carrot and topinambur, compared to other vegetable peels.

**Fig 2:**
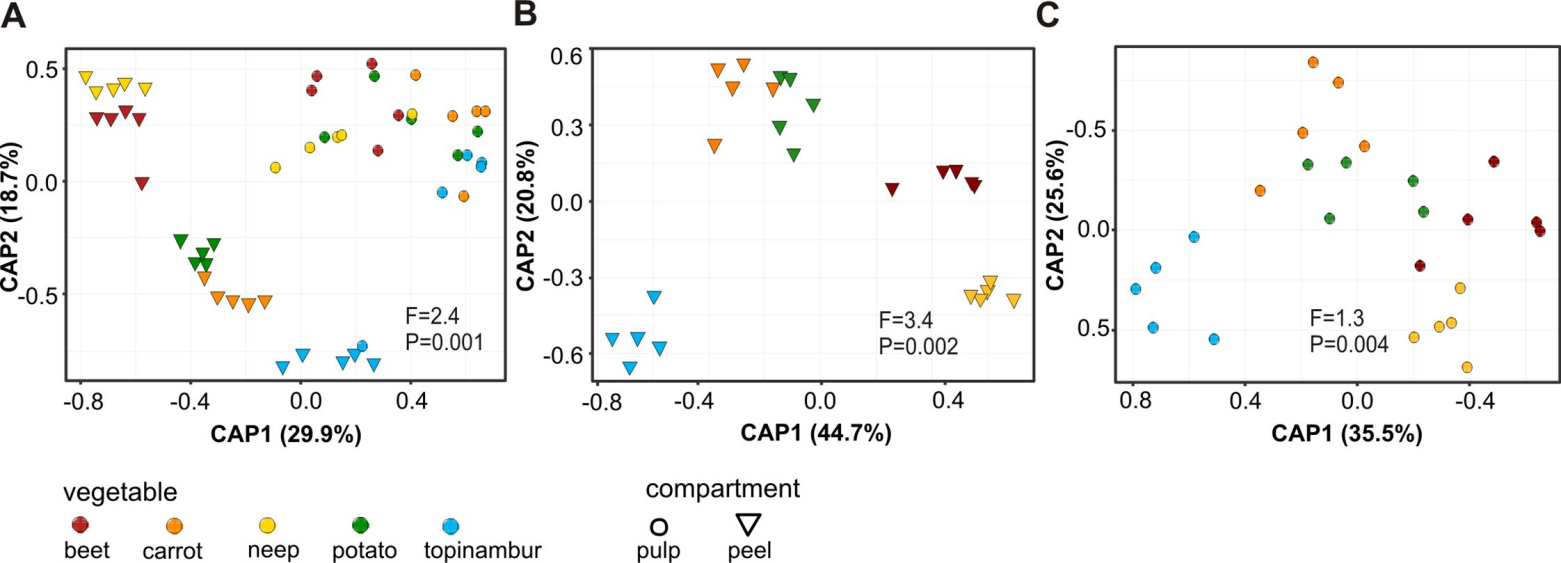
Bacterial community structure of five root vegetables. Bray–Curtis distances and Constrained Analysis of Principal Coordinates (CAP) of OTUs were performed for both vegetable compartments (peel/pulp) and each vegetable species. (A) CAP constrained by compartments and vegetable species. (B) CAP of vegetable peels constrained by vegetable species. (C) CAP of vegetable pulps constrained by vegetable species. Both the F-statistic and P-values are shown.

To analyze the influence of sample origin (peel/pulp and vegetables species) on the microbiome diversity we performed a Constrained Analysis of Principal Coordinates (CAP) of the Bray-Curtis distance of OTU abundances (Fig 2). The peel and pulp microbiomes were clearly separated on the CAP plot (Fig 2A). The peel microbiomes were clustered by plant species, whereas in case of the pulp microbiomes this effect is much less pronounced (Fig 2A). To further analyze the distribution of bacterial communities between different vegetables we performed another CAP analysis for the vegetable compartments. Fig 2B and 2C show that the microbiomes of vegetable peels form well-defined clusters, while the microbiomes of pulps do not separate well between the vegetables, with the exception of topinambur. This suggests that the peel microbiomes of different vegetables are more different from each other than the pulp microbiomes.

To test the effects of vegetable compartments and the vegetable species on their microbiomes, we used PERMANOVA. According to the analysis, the pairwise distances between microbial communities differed significantly between the peel and the pulp (F = 6.34, R^2^ = 0.096, P < 0.001), and between different vegetables (F = 3.26, R^2^ = 0.198, P < 0.001). There is a significant difference between vegetable peels (F = 5.514, R^2^ = 0.52444, P < 0.001), however, we observe no significant difference between vegetable pulps (F = 1.2772, R^2^ = 0.20346, P = 0.075). We conclude that the formal PERMANOVA analysis is in good agreement with the exploratory visual representation of CAP.

### Microbial taxonomic composition

**Fig 3:**
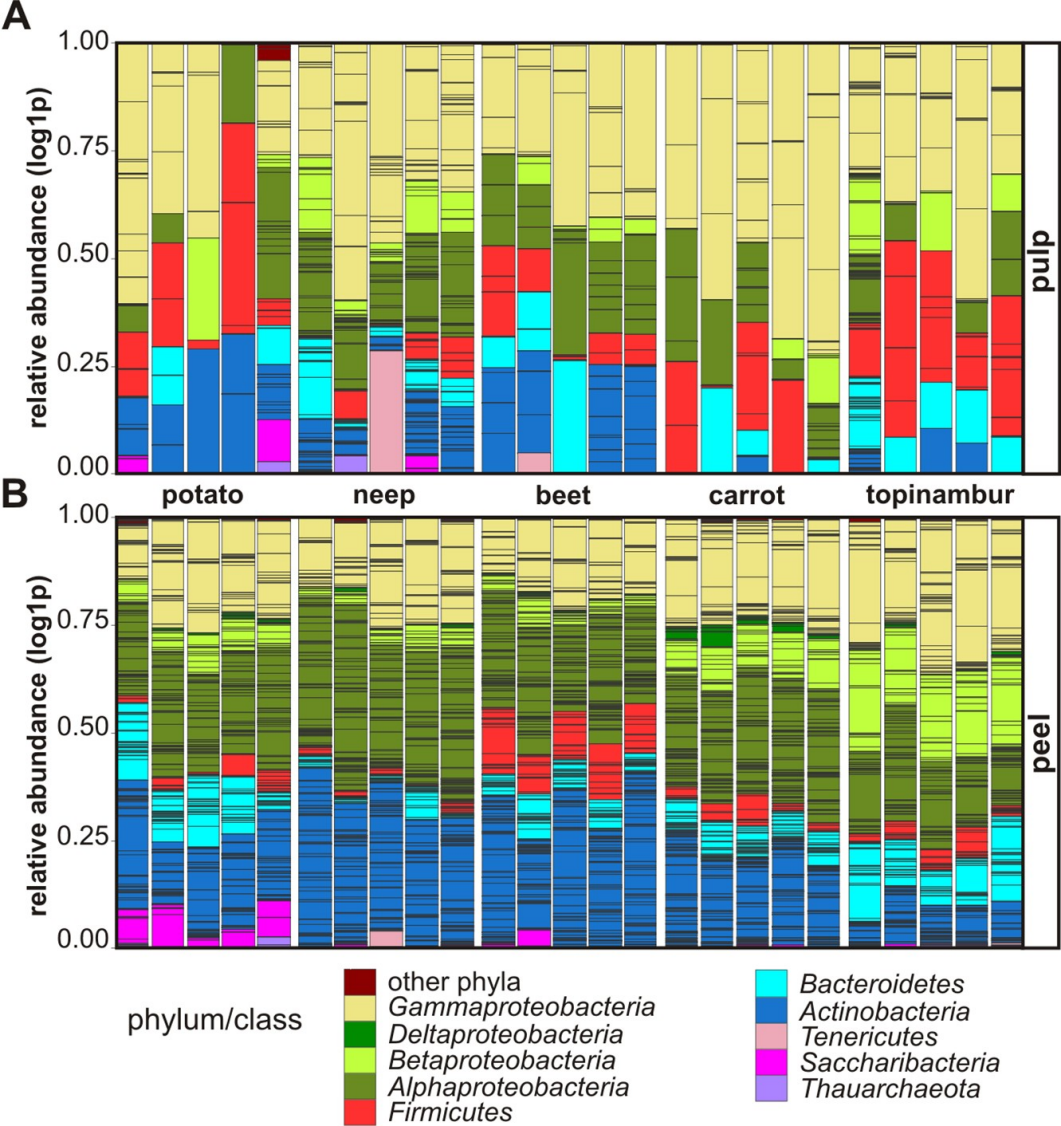
Log-transformed relative abundances of bacterial phyla found in five root vegetables. All OTUs defined in the current study are indicated on the bar based on a phylum/class color code. *Proteobacteria* are shown at the class level, other bacteria at the phylum level. Pulp (A) and peel (B) are shown.

In order to describe the taxonomic structure of microbial communities in vegetables, we studied the relative abundances of phyla and larger classes (Fig 3). Altogether, we detected sequences from 15 phyla–*Thauarcheota (Archea)*, *Saccharibacteria* (TM7), *Tenericutes*, *Actinobacteria*, *Bacteroidetes*, *Firmicutes*, *Proteobacteria*, *Armatimonadetes*, *Chlamydiae*, *Chloroflexi*, *Gemmatimonadetes*, *Planctomycetes*, *Verrucomicrobia*, *Gracilibacteria* and *Deinococcus-Thermus*. In all vegetable peels *Actinobacteria*, *Alphaproteobacteria* and *Gammaproteobacteria* were distributed quite evenly. *Betaproteobacteria* were abundant in carrot and topinambur, *Firmicutes* in beet, and *Saccharibacteria* in potato.

To determine which bacterial phyla differentiate the microbiomes of a particular vegetable, we visualized the members of dominant phyla/classes on a bar plot and performed another CAP analysis (S1 Fig). The phylogenetic pattern of phylum *Actinobacteria* most clearly differentiates the peels of neep and beet from other vegetable samples. The microbiome of topinambur peels, which has fewer OTUs, was assigned to the phylum *Actinobacteria* cluster together with the microbiomes of all vegetable pulps. OTUs from the class *Alphaproteobacteria*, mostly from the order *Rhizobiales*, clearly differentiate the microbiomes of peels and pulps. The number of OTUs classified as *Alphaproteobacteria* is clearly more abundant in the microbiomes of all vegetable peels than in pulp. Although not evenly distributed, OTUs assigned to the phylum *Firmicutes* are more abundant in the microbiomes of pulps than in peels. OTUs classified within the family *Enterococcaceae* tend to be specific to topinambur pulp. The CAP analysis revealed that class *Betaproteobacteria* was found to associate more with the microbiome of topinambur peels compared with other vegetables, in accordance with the relative abundances of families: samples of the microbiome of topinambur peel contain ca 15% *Oxalobacteraceae*, while the other peel microbiomes only 3–5%. OTUs from the phylum *Saccharibacteria* are characteristic for potato. OTUs assigned to the class *Gammaproteobacteria* are relatively more abundant and more evenly distributed in all vegetable species and compartments.

### Core OTUs of five root vegetables

**Fig 4:**
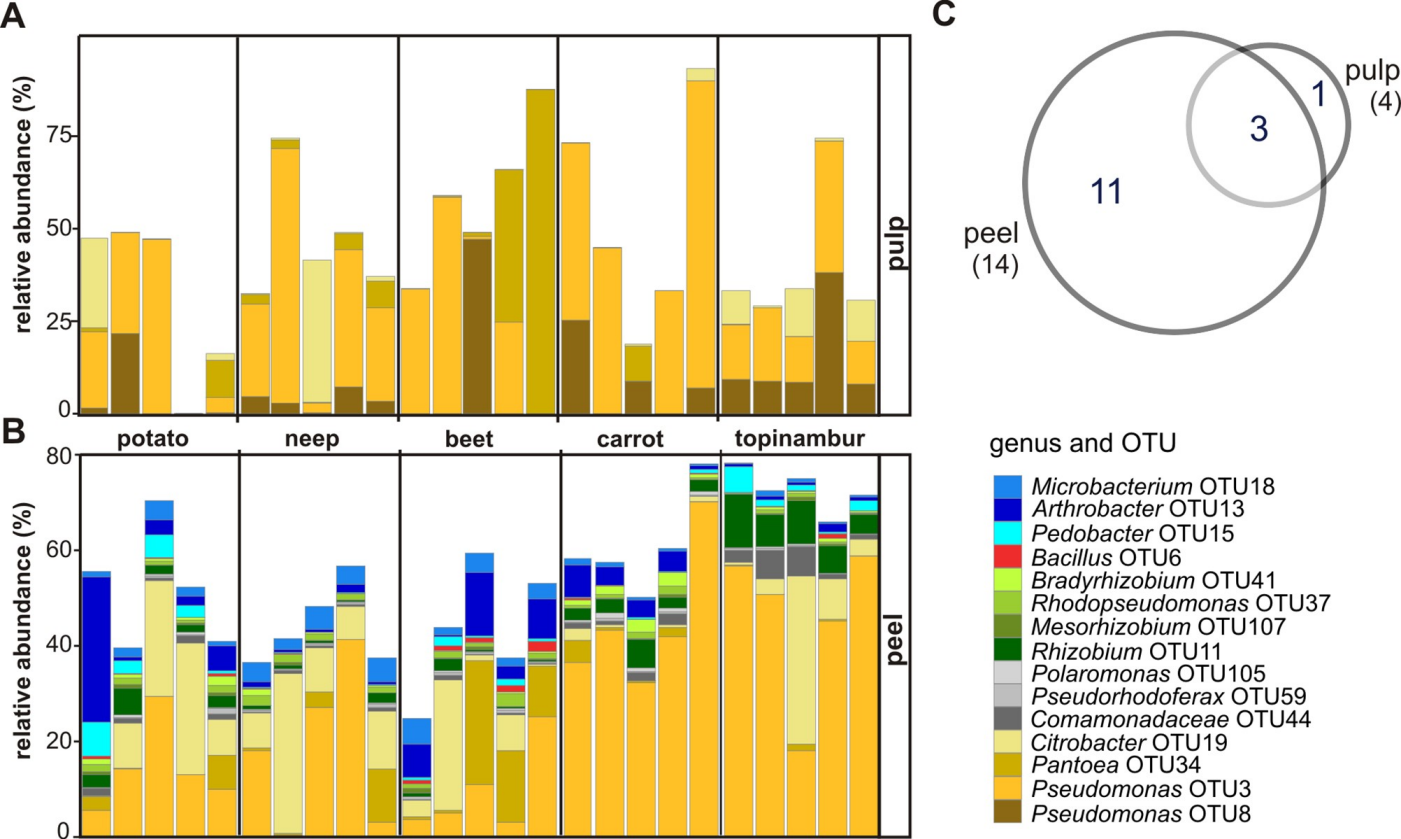
Relative sequence abundances of the core OTUs shared in the datasets of five root vegetables. The core OTUs of pulp (A) and peel (B) are shown. A Venn diagram shows the number of core OTUs shared between peel and pulp (C).

Abundances of core OTUs, that is OTUs present in all samples of vegetable peels or all samples of pulp, are shown in a Fig 4A and 4B. There are fourteen OTUs common to all peels of five vegetables, with the most abundant being *Proteobacteria*. Only four OTUs were common in the pulps of all five root vegetables, three of which also belong to the core microbiome of vegetable peels (Fig 4C). The only OTU characteristic to the pulp of all five root vegetables is *Pseudomonas sp*. OTU8. Core OTUs of both the peel and the pulp belong to the class *Gammaproteobacteria*: *Citrobacter* OTU19, *Pantoea* OTU34 and *Pseudomonas* OTU3. *Pseudomonas* OTU3 is the most abundant both in vegetable peels and pulps. It makes up most of the total microbiome of the peels of carrot (ca 40%) and topinambur (ca 50%), thus explaining the low Shannon diversity value for these vegetable samples (Fig 1).

### Characteristic OTUs

**Fig 5:**
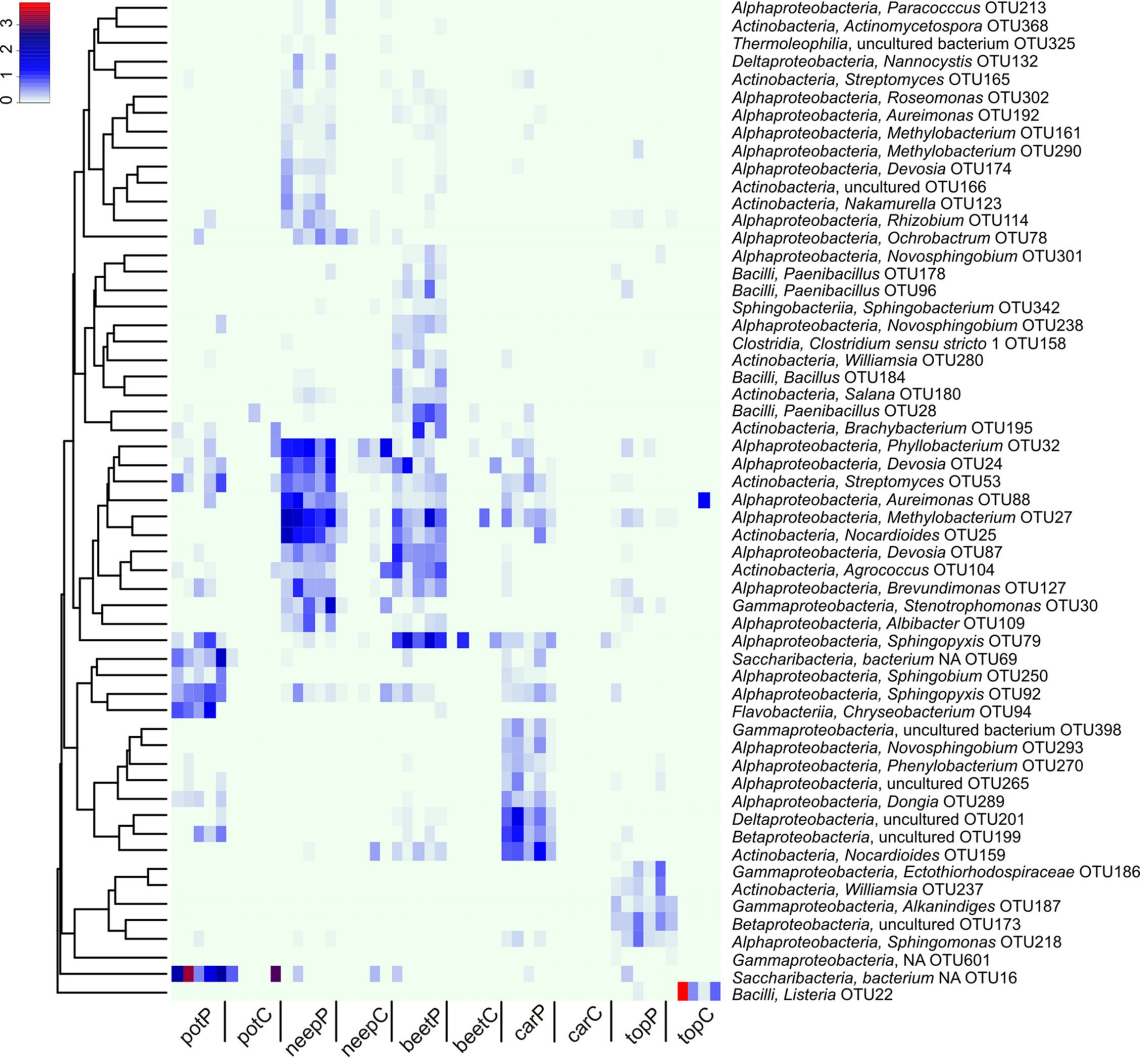
Species that are characteristic to the peel and pulp of five root vegetables. Relative abundances of OTUs specific to only one vegetable compartement (P- value <0.001) were clustered using Bray-Curtis dissimilarity and depicted on a heatmap. The class and genus of the OTUs are indicated. Abbreviations: neepC–neep pulp; neepP–neep peel; carC–carrot pulp; carP–carrot peel; topC–topinambur pulp; topP–topinambur peel; potC–potato pulp; potP–potato peel; beetC–beet pulp; beetP–beet peel. We included only those OTUs whose read counts in all samples was more than 50 in the analysis.

In order to investigate the bacterial species/OTUs characteristic to a particular vegetable we used indicator species analysis [46]. Relative sequence abundances of OTUs that were significantly correlated with a particular vegetable peel or pulp (p ≤0.001) were clustered using Bray-Curtis dissimilarity. Fig 5 shows the OTUs that specifically represent only the peel of neep, beet, carrot, topinambur, or potato. In accordance with the CAP analyses (Fig 2), there are OTUs characteristic to the peels of both neep and beet or potato and carrot. In contrast to peels, there was only one indicative OTU (*Listeria sp*. OTU22) associated with the pulp of topinambur. Pulps of other vegetable species have no OTUs that exhibit an association at the significance level ≤ 0.001.

Taken together, the results of the core and the characteristic species studies indicate that the peels of vegetables have several common OTUs, as well as OTUs that distinguish different vegetable species, while there is only one OTU common to all pulps of vegetable roots. Only topinambur pulp has a characteristic OTU.

### Cultivated bacteria

Dilutions of the unfrozen samples were plated on R2A agar and on FAA for the cultivation and CFU counting of bacteria. Dominant groups of bacteria, as determined by MALDI-TOF fingerprinting, are shown in S1 Table. Because many bacterial groups are difficult to cultivate, a high correlation between sequencing and cultivation results should not be expected.

The dominant genus *Pseudomonas*, although well cultivable, is under-represented in our cultivation assay, whereas the genera *Bacillus* and *Paenibacillus* are over-represented by cultivation, as compared to sequencing. *Bacillus* and *Paenibacillus* are spore-formers and therefore might have better survived the freezing-melting procedures of our study. In addition, it has been previously described that these genera are underestimated by the PCR-based next-generation sequencing, as compared to cultivation [32,49].

The pulp of potato 4 was dominated by *Paenibacillus polymyxa*, as determined by either method. During cultivation, it was observed that *P*. *polymyxa* suppressed the growth of other bacteria on the cultivation plate. The ability to inhibit other bacteria might indicate a mechanism for taking over the community inside the vegetable (note the lack of otherwise dominating *Pseudomonas* OTU3 in potato 4, Fig 4).

**Fig 6:**
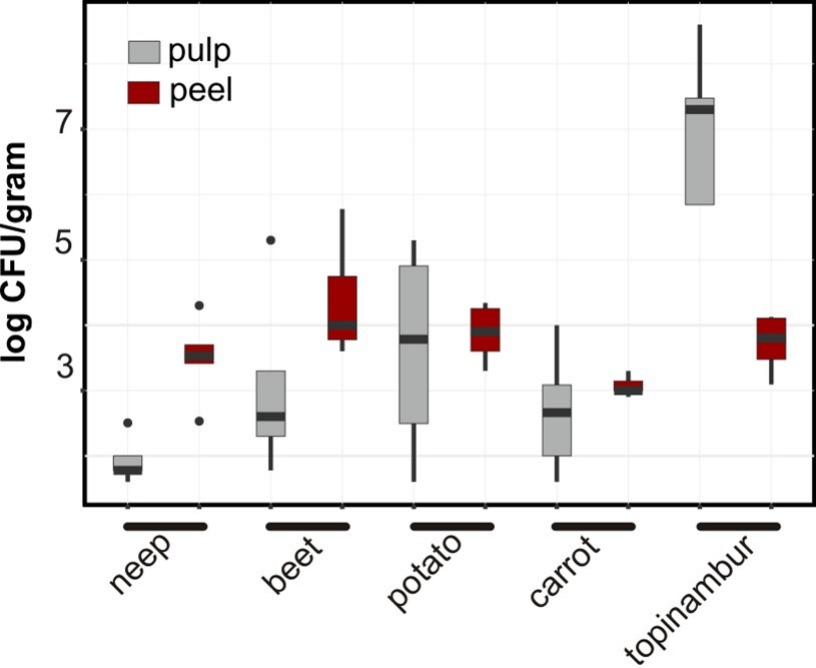
Median CFU counts of cultivated bacteria. Dilutions of the melted samples for CFU counting were plated on both R2A agar and FAA and incubated for one week at room temperature.

In general, the CFU counts were higher in samples from peels (Fig 6). One interesting exception is topinambur, which contained higher CFU counts in samples from the pulp (~10^6^–10^8^ colonies/gram) compared to the peel (~10^4^ colonies/gram). The inner community of topinambur contains a considerable number of *Carnobacterium maltoaromaticum* and *Enterococcus faecalis* species, thus indicating anaerobic fermentation of the plant tissue.

## Discussion

Plants and animals harbor specific bacterial communities shaped by the host immune system, whereas resident bacteria profoundly shape host immunity [50–55]. Still, the host microbial communities are complex and dynamic ecosystems in which species are in continuous flux [55,56].

We propose that food microbiomes have an impact on the human microbiome and immune system. Therefore we choose vegetable roots that could be eaten raw in order to study their bacterial content. We choose five root vegetables from different plant families that are traditionally stored at low temperatures (around +4 to +15°C) during the period of no vegetation. Usually, in microbiome studies the root is dissected into three compartments: rhizosphere, rhizoplane, and endosphere. The aim of our study was to investigate the bacterial communities that are present in the vegetables that are consumed as raw food. Potatoes are not commonly eaten raw, but still several recipes with raw potatoes are getting more attention.

We used standard food processing technics to separate vegetable compartments, instead of the methods currently used to study plant roots [3]. The compartment we analyzed as peels consisted of mainly rhizoplane, however, considering our isolation techniques, it also contains traces of rhizosphere and endosphere. The pulp of root vegetables in our study should provide pure endosphere comparable to other studies.

We observed similar trends as in many previous studies, the dominant bacterial phyla in all plant root compartments are often the same with the most abundant being *Proteobacteria*, followed by *Actinobacteria*, *Bacteroidetes*, and *Firmicutes* [29,36,51,57,58]. The ratio between these phyla and the presence of minor phyla depend on the plant genotype, growth stage and environment [59–62].

We observed that the class *Alphaproteobacteria* distinguishes peel and pulp samples of all vegetable studied. The main characteristic features of microbiomes are related to the peel (Fig 2). In our study, potato, neep, beet, and carrot were grown in the same farm on adjacent fields and kept in the same cellar, suggesting that the differences in bacterial composition are attributable to the genetic nature of each plant: firstly, the microbiota of neep and beet peels tend to have higher richness and diversity than the other vegetable peels (Fig 1). In peels the differentiating bacteria belong to the phylum *Actinobacteria*, a common group of soil bacteria (S1 Fig). The underlying reason may be that neep and beet have thicker and denser peels. Secondly, the peel of potato contains considerable amounts of *Saccharibacteria* lacking in other vegetables (Figs 3 and 4, S1 Fig). Relatively high numbers of *Saccharibacteria* in potato has been noted previously [29,32]. Thirdly, the phylum *Firmicutes* is relatively more abundant in the peel of beet than in the other vegetable peels (Figs 3, 4 and 5).

Topinambur was most clearly separated from the other root vegetables in the CAP analysis (Fig 2). This may be a specific characteristic of the species, however, because its samples originated from another district of Estonia, the influence of the soil cannot be excluded.

The microbiome is believed to be gradually enriched from the bulk soil inoculum [3,63–65]. It is then presumptive that the bacteria present in the pulp of vegetables are selectively derived from the peel. We found that there are considerably fewer bacterial species/OTUs in the pulps of vegetable roots, and that the CFU count of bacteria is also 1–2 orders of magnitude lower compared to the peels. This might explain the heterogeneous distribution of bacteria between samples of pulps in our study: by chance, PCR amplifies the handful of detected bacterial sequences among a huge number of plastid and mitochondrial sequences present in the plant tissue. Still, it is clear that the most distinguishing feature of vegetable pulp is the high fraction of *Gammaproteobacteria*.

Although we chose visibly healthy vegetables for our study, we found evidence that the plant decay processes can start from the inner anoxic part of the vegetable. This process was most clearly seen in case of topinambur, although they remained crunchy and no spoilage was observed according to the taste and smell. The CFU count of the pulp of topinambur was 3–4 magnitudes higher than its peel. The high number of CFUs was caused by *Enterococcus faecalis* and *Carnobacterium maltoaromaticum*. *Enterococcus* and *Carnobacteria* are ubiquitous lactic acid bacteria (LAB) able to colonize several ecological niches, including vegetables, meat, dairy substrates, and the gastro-intestinal tract [66,67]. It has to be noted that the field, where topinambur was grown, was fertilized with manure several years ago. This might have contributed to the soil microbiome, which in turn influences the microbial composition of the vegetables. Therefore, the influence of soil and fertilizer on the amounts of *Enterecocci* and *Lactobacteria* in vegetable need to be carefully considered in future studies.

In growing plants, the total amount of the order *Lactobacillales* is usually under 0.1% of the relative abundance of microbial composition [53]. Thus, LAB, which belonging to order *Lactobacillales*, are mostly under the detection level in growing plants, but can increase in number under anaerobic conditions when the decomposition of plant material starts. This phenomenon is widely used to conserve plant material for making silage or for fermenting pickles. The drop in pH, which results from the production of lactic and acetic acid by LAB, protects the plant material from spoilage [68]. The genus *Enterococcus* is a controversial group of lactic acid bacteria. Although, some members of the group have been described as nosocomial pathogens, some members are used as probiotics [69–71]. Strains belonging to the genus *Enterococcus* produce a wide variety of bacteriocins active against Gram-positive foodborne pathogens [72].

Why the plant decay in topinambur starts with LAB instead of bacteria that produce plant cell wall-degrading enzymes is unclear. One explanation might be low temperature: both *Carnobacterium* and *Enterococcus* can grow at low temperatures [73]. Indeed, the topinambur tubers were harvested in January from frozen soil. It is common practice to keep topinambur tuber unharvested in the soil because the vegetable can stand low temperatures due to cryoprotection by inulin.

Most studies about plant root microbiome are focused on agriculturally important plants such as rice [3,60], wheat [74], maize [75], and soya [74], however, these plants are not consumed raw. Less is known about the plant microbiome at the point of raw consumption, excepting studies that focus on the above-soil part of the plant and on the occurrence of potential pathogens [25,76–78]. Considering the composition of microbiota of root vegetables, it is probable that consuming these vegetables as raw food has an impact on human health. For example, it has been shown that the diversity of bacterial communities we come into contact with influences the development of our immune system [79,80]. As the diversity and number of bacteria is considerably higher in peels than in the pulp of root vegetables, extensive peeling diminishes the potential beneficial impact on our immune system. In addition to the potential beneficial effects of plant microbiota we should also carefully consider the pathogenic potential to humans. For example, in the pulp of topinambur genus we observed *Listeria* (Fig 5). Although 16S rDNA does not discriminate between pathogenic and non-pathogenic species, this food-safety aspect ought to be considered.

In this study we observed a large variation between samples; therefore a thorough characterization of root vegetable microbiome would require larger studies that cover different varieties, soils, and storage conditions (temperature, humidity). Careful research has to be performed over several years involving several sampling times. In addition, it would be important to compare the influence of organic farming and intensive farming on each plants microbiome.

## Supporting information

S1 Fig Detailed analyses of the most abundant phyla/classes of vegetable microbiomes. The members of dominant phyla/classes of vegetable microbiomes are visualized on barplots by the color code based on the microbial order (phylum *Actinobacteria*, class *Alphaproteobacteria*), class (phylum *Saccharibacteria*) or family (other phyla and classes). Constrained Analysis of Principal Coordinates (CAP) of OTUs assigned to major microbial phyla detected in vegetables. The ANOVA statistic was applied to the ordinations; corresponding F- and P-values are shown. Only OTUs whose read counts in all samples were more than 50 were studied.
(TIF)

S1 Table Dominant cultivated bacteria and dominating OTUs in five root vegetables. Top three dominant cultivated bacteria in each vegetable sample based on MALDI-TOF mass spectrometry identification and top three dominating OTUs from the same samples indicated in the adjacent column. Abbreviations: neepC–neep pulp; neepP–neep peel; carC–carrot pulp; carP–carrot peel; topC–topinambur pulp; topP–topinambur peel; potC–potato pulp; potP–potato peel; beetC–beet pulp; beetP–beet peel.
(DOCX)
