# Supplementary figures and images for: Endophytic bacterial communities in peels and pulp of five root vegetables
Source: PLoS One. 2019 Jan 11;14(1):e0210542. doi: 10.1371/journal.pone.0210542 (PMC6329509; doi:10.1371/journal.pone.0210542)

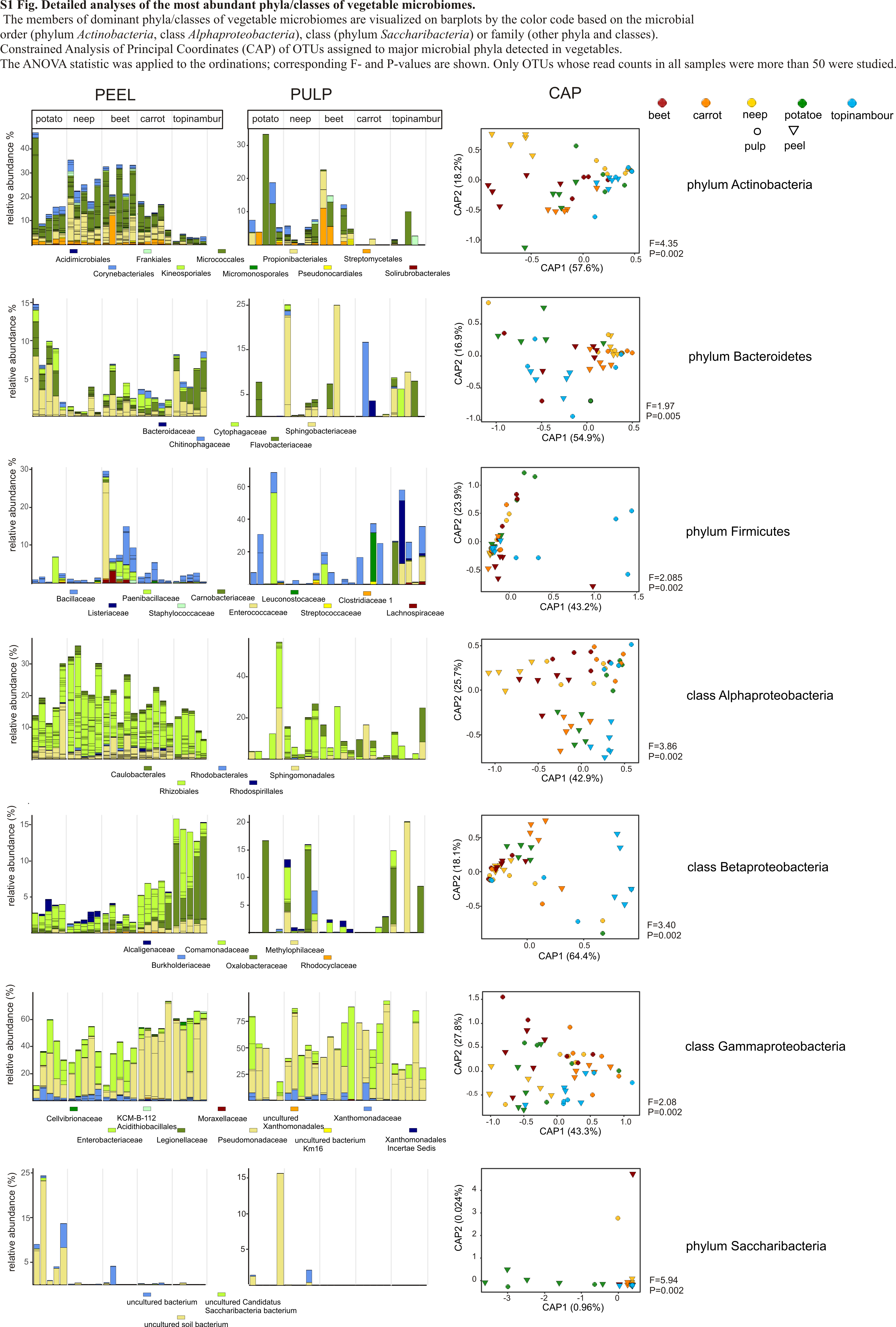

Supplement: S1 Fig — The members of dominant phyla/classes of vegetable microbiomes are visualized on barplots by the color code based on the microbial order (phylum Actinobacteria, class Alphaproteobacteria), class (phylum Saccharibacteria) or family (other phyla and classes). Constrained Analysis of Principal Coordinates (CAP) of OTUs assigned to major microbial phyla detected in vegetables. The ANOVA statistic was applied to the ordinations; corresponding F- and P-values are shown. Only OTUs whose read counts in all samples were more than 50 were studied. (TIF) [file pone.0210542.s001.tif]
