# Supplementary material for: Endophytic bacterial communities in peels and pulp of five root vegetables
Source: PLoS One. 2019 Jan 11;14(1):e0210542. doi: 10.1371/journal.pone.0210542 (PMC6329509; doi:10.1371/journal.pone.0210542)
Supplement: S1 Table — Top three dominant cultivated bacteria in each vegetable sample based on MALDI-TOF mass spectrometry identification and top three dominating OTUs from the same samples indicated in the adjacent column. Abbreviations: neepC–neep pulp; neepP–neep peel; carC–carrot pulp; carP–carrot peel; topC–topinambur pulp; topP–topinambur peel; potC–potato pulp; potP–potato peel; beetC–beet pulp; beetP–beet peel. (DOCX) [file pone.0210542.s002.docx]

**S1 Table. Dominant cultivated bacteria and dominating OTUs in five root vegetables.**

Top three dominant cultivated bacteria in each vegetable sample based on MALDI-TOF mass spectrometry identification and top three dominating OTUs from the same samples indicated in the adjacent column. Abbreviations: neepC – neep pulp; neepP – neep peel; carC – carrot pulp; carP – carrot peel; topC – topinambur pulp; topP – topinambur peel; potC – potato pulp; potP – potato peel; beetC – beet pulp; beetP – beet peel.

| **vegetable** | **CFU** | **cultivated,**  **MALDI identification** | **genus OTU,**  **llumina sequencing** |
| --- | --- | --- | --- |
|  |  |  |  |
| **potP1** | 2,00E+03 | *Bacillus muralis, Cellulomonas sp.* | *Arthrobacter* OTU13, *Pedobacter* OTU15,  *Saccharibacteria* OTU16 |
| **potP2** | 2,2E+04 | *Microbacterium sp., Bacillus muralis,*  *Bacillus lichenoformis* | *Saccharibacteria* OTU16, *Pseudomonas* OTU3, *Citrobacter* OTU19 |
| **potP3** | 1,8+04 | *Paenibacillus, Bacillus mycoides,*  *Bacillus lichenoformis* | *Pseudomonas* OTU3, *Citrobacter* OTU19,  *Stenotrophomonas* 14 |
| **potP4** | 8,00E+03 | *Paenibacillus, Paenibacillus polymyxa,*  *Bacillus lichenoformis* | *Citrobacter* OTU19, *Pseudomonas* OTU3,  *Paenibacillus* OTU5 |
| **potP5** | 4,00E+03 | *Staphylococcus epidermidis, Paenibacillus polymyxa, Bacillus lichenoformis* | *Sphingobium* OTU181, *Pseudomonas* OTU3,  *Saccharibacteria* OTU18 |
| **neepP1** | 3,40E+03 | *Psychrobacillus sp., Bacillus mycoides,*  *Viridibacillus sp.* | *Pseudomonas* OTU3, *Microbacterium* OTU12,  *Citrobacter* OTU19 |
| **neepP2** | 2,60E+03 | *Bacillus muralis, Bacillus megaterium,*  *Bacillus dretensis* | *Citrobacter* OTU19, *Microbacterium* OTU12,  *Methylobacterium* OTU27 |
| **neepP3** | 2,00E+04 | *Paenibacillus odorifer, Bacillus megaterium, Bacillus pumilus* | *Pseudomonas* OTU3, *Citrobacter* OTU19,  *Microbacterium* OTU12 |
| **neepP4** | 5,00E+03 | *Paenibacillus odorifer, Bacillus megaterium, Bacillus simplex* | *Pseudomonas* OTU 3*, Citrobacter* OTU19,  *Microbacterium* OTU12 |
| **neepP5** | 3,40E+02 | *Bacillus muralis, Bacillus mycoides,*  *Paenibacillus polymyxa* | *Citrobacter* OTU19, *Pantoea* OTU34,  *Sanguibacter* OTU23 |
| **beetP1** | 5,60E+04 | *Klebsiella oxytoca, Staphylococcus sp.,*  *Carnobacterium maltaromaticum* | *Enterococcus* OTU9, *Arthrobacter* OTU13,  *Microbacterium* OTU18 |
| **beetP2** | 4,00E+03 | *Bacillus muralis, Bacillus weichenstephanensis, Bacillus simplex,* | *Citrobacter* OTU19, *Streptomyces* OTU19,  *Sphingopyxis* OTU79 |
| **beetP3** | 6,00E+05 | *Pantoea agglomerans, Lactobacillus amylovorus, Psychrobacillus sp.* | *Pantoea* OTU34, *Arthrobacter* OTU13,  *Pseudomonas* OTU3 |
| **beetP4** | 6,00E+03 | *Bacillus megaterium, Bacillus muralis,*  *Psychrobacillus sp.* | *Pantoea* OTU34, *Bacillus* OTU17,  *Citrobacter* OTU19 |
| **beetP5** | 1,00E+04 | *Bacillus mycoides, Bacillus megaterium,*  *Paenibacillus mendelii* | *Pseudomonas* OTU3, *Pantoea* OTU34,  *Arthrobacter* OTU34 |
| **carP1** | 2,00E+03 | *Pantoea agglomerans,*  *Bacillus megaterium* | *Pseudomonas* OTU3, *Pseudomonas* OTU8,  *Arthrobacter* OTU13 |
| **carP2** | 1,00E+03 | *Bacillus megaterium, Bacillus mycoides,*  *Bacillus drentensis* | *Pseudomonas* OTU3, *Pseudomonas* OTU8,  *Arthrobacter* OTU13 |
| **carP3** | 8,00E+02 | *Psychrobacillus sp., Bacillus mycoides,*  *Bacillus simplex* | *Pseudomonas* OTU3, *Pseudomonas* OTU8,  *Rhizobium* OTU11 |
| **carP4** | 1,00E+03 | *Paenibacillus amylolyticus,*  *Bacillus mycoides* | *Pseudomonas* OTU3, *Pseudomonas* OTU8,  *Arthrobacter* OTU13 |
| **carP5** | 1,40E+03 | *Solibacillus sp., Lysinibacillus sp.,*  *Bacillus megaterium* | *Pseudomonas* OTU3, *Janthinobacterium* OTU21, *Rhizobium* OTU11 |
| **topP1** | 1,24E+03 | *Bacillus simplex, Psychrobacillus sp.,*  *Bacillus weichenstephanensis* | *Pseudomonas* OTU3, *Rhizobium* OTU11, *Janthinobacterium* OTU21 |
| **topP2** | 3,00E+03 | *Ewingella sp., Bacillus simplex,*  *Pantoea agglomerans* | *Pseudomonas* OTU3, *Rhizobium* OTU11,  *Comamonadaceae* OTU44 |
| **topP3** | 1,28E+04 | *Bacillus simplex,*  *Bacillus weichenstephanensis* | *Citrobacter* OTU19, *Pseudomonas* OTU3,  *Rhizobium* OTU11 |
| **topP4** | 1,34E+04 | *Serratia liquenfaciens,*  *Bacillus simplex* | *Pseudomonas* OTU3, *Pseudomonas* OTU8,  *Citrobacter* OTU19 |
| **topP5** | 6,40E+03 | *Psychrobacillus sp.,*  *Bacillus simplex* | *Pseudomonas* OTU3, *Janthinobacterium* OTU21, *Rhizobium* OTU11 |
| **potC1** | 2,00E+01 | *Staphylococcus capitis* | *Escherichia-Shigella* OTU45, *Citrobacter* OTU19, *Pseudomonas* OTU3 |
| **potC2** | 6,00E+04 | *Pseudomonas rhodesiae* | *Pseudomonas* OTU3, *Pseudomonas* OTU8,  *Aeribacillus* OTU80 |
| **potC3** | 2,00E+05 | *Pseudomonas extremorientalis* | *Pseudomonas* OTU3, *Terrabacter* OTU177, *Janthinobacterium* OTU21 |
| **potC4** | 4,00E+01 | *Paenibacillus polymyxa* | *Paenibacillus* OTU5, *Aeribacillus* OTU80,  *Ancylobacter* OTU172 |
| **potC5** | 6,20E+02 | *Bacillus megaterium, Bacillus subtilis* | *Bradyrhizobium* OTU41, *Sphingomonas* OTU33, *Saccharibacteria* OTU16 |
| **neepC1** | 6,00E+01 | *Bacillus megaterium* | *Pseudomonas* OTU3, *Pedobacter* OTU15,  *Stenotrophomonas* OTU14 |
| **neepC2** | 1,00E+02 | *Serratia sp., Enterobacter sp.,*  *Stenotrophomonas sp.* | *Pseudomonas* OTU3, *Stenotrophomonas* OTU14, *Labrys* OTU43 |
| **neepC3** | 6,00E+01 | *Oerskovia sp.,*  *Bacillus megaterium* | *Candidatus Phytoplasma* OTU35, *Citrobacter* OTU19, *Pseudomonas* OTU3 |
| **neepC4** | 4,00E+01 | *Paenibacillus sp.* | *Pseudomonas* OTU3, *Rhizobium* OTU12, *Janthinobacterium* OTU21 |
| **neepC5** | 3,20E+02 | *Lysinibacillus, Debaryomyces hansenii, Staphylococcus warneri* | *Pseudomonas* OTU3, *Bradyrhizobium* OTU41,  *Pantoea* OTU34 |
| **beetC1** | 2,00E+05 | *Pseudomonas extremorientalis, Pseudomonas veronii* | *Pseudomonas* OTU3, *Paenibacillus* OTU5,  *Streptomyces* OTU39 |
| **beetC2** | 2,00E+02 | *Pseudomonas sp., Microbacterium saperdae ,*  *Corynebacterium pseudodiphtheriticum* | *Pseudomonas* OTU3, Aeribacillus OTU80,  *Microbacterium* OTU18 |
| **beetC3** | 2,00E+03 | *Pseudomonas grimontii* | *Pseudomonas* OTU8, *Rhizobium* OTU12, *Pedobacter* OTU15 |
| **beetC4** | 4,00E+02 | *Pantoeae agglomerans*,  *Serratia* *sp.* | *Pantoea* OTU34, *Pseudomonas* OTU3,  *Arthrobacter* OTU13 |
| **beetC5** | 6,00E+01 | *Lelliottia amnigena,*  *Pantoea agglomerans* | *Pantoea* OTU34, *Aeromicrobium* OTU29  *Caulobacteraceae* OTU58 |
| **carC1** | 1,00E+02 | *Pseudomonas extremorientalis* | *Pseudomonas* OTU3, *Pseudomonas* OTU8,  *Bacillus* OTU6 |
| **carC2** | 4,00E+01 | *Pseudomonas fluoresscens* | *Pseudomonas* OTU3, *Sphingomonas* OTU33, *Ferruginibacter* OTU221 |
| **carC3** | 4,60E+02 | *Rahnella aquatilis* | *Leuconostoc* OTU10, *Escherichia-Shigella* OTU45, *Pantoea* OTU34 |
| **carC4** | 1,00E+04 | *Pseudomonas lundensis* | *Pseudomonas* OTU3, *Escherichia-Shigella* OTU45, *Bacillus* OTU6 |
| **carC5** | 1,22E+03 | *Serratia liquenfaciens,*  *Methylobacterium* sp. | *Pseudomonas* OTU3, *Pseudomonas* OTU8,  *Citrobacter* OTU8 |
| **topC1** | 2,00E+07 | *Carnobacterium maltoaromaticum* | *Carnobacterium* OTU36*, Pseudomonas* OTU3,  *Rhizobium* OTU11 |
| **topC2** | 3,00E+07 | *Serratia plymuthica, Pseudomonas grimontii,*  *Enterococcus faecalis* | *Listeria* OTU22, *Pseudomonas* OTU3,  *Enterococcus* OTU9 |
| **topC3** | 4,00E+08 | *Lelliottia amnigena,*  *Enterococcus faecalis* | *Methylotenera* OTU102, *Enterococcus* OTU9,  *Citrobacter* OTU19 |
| **topC4** | 7,00E+05 | *Ewingella sp.,*  *Pseudomonas extremorientalis,* | *Pseudomonas* OTU8, *Pseudomonas* OTU3,  *Escherichia-Shigella* OTU45 |
| **topC5** | 7,00E+05 | *Pseudomonas veronii, Carnobacterium maltoaromaticum, Enterobacter sp.* | *Enterococcus* OTU9, *Aeribacillus* OTU80,  *Caulobacteraceae* OTU58 |
